# Movement of the sacroiliac joint during a modified active straight leg raise measured with CT-RSA: a feasibility study

**DOI:** 10.2340/17453674.2026.45479

**Published:** 2026-03-03

**Authors:** Vinjar Brenna HANSEN, Anselm SCHULZ, Christian HELLUM, Stephan M RÖHRL

**Affiliations:** 1 Haukeland University Hospital, Klinisk institutt 1, Bergen; 2 Orthopedic Department, Oslo University Hospital, Rikshospitalet, Oslo; 3 Division of Radiology and Nuclear Medicine, Oslo University Hospital, Ullevål, Oslo; 4 Orthopedic Department, Oslo University Hospital, Ullevål, Oslo; 5 University of Oslo, Institute for Clinical Medicine, Oslo, Norway

RSA is the established gold standard for measuring the movement of implants and across joints [1]. CT-RSA without RSA markers has emerged as a potential new method for measuring in vivo motion of the sacroiliac joint (SIJ) [2-4]. The placement of RSA markers is invasive and costly and not needed for CT-RSA. We aimed to clarify whether CT-RSA can measure in vivo movement in the SIJ during a modified active straight leg raise (ASLR). To control and compare the results, we also performed conventional RSA in these patients.

## Methods

**Figure 1:**
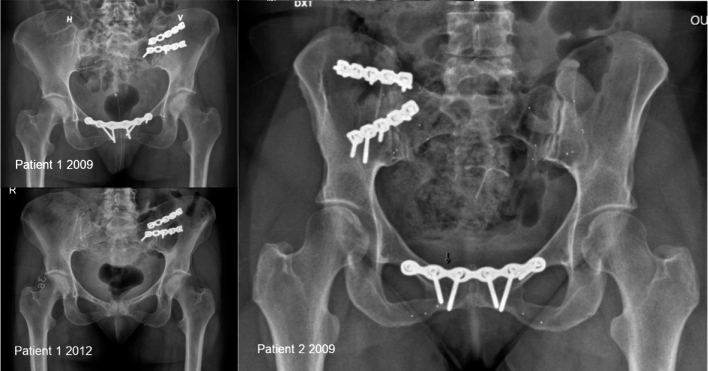
Left: Patient 1, aged 45, had the left sacroiliac joint (SIJ) and symphysis plate-fixated in 2009. The plate fixation of the symphysis was removed in 2012. She now has recurrent pelvic girdle pain. Right: Patient 2, aged 60, had the right SIJ and symphysis plate-fixated in 2009 and improved clinically thereafter, but now has recurrent pelvic girdle pain.

We recruited 2 patients from a former study on chronic pelvic girdle pain at Oslo University Hospital [5], for whom SIJ fusion was planned because of recurrent pain (Figure 1). Both patients (female) had an arthrodesis performed on 1 side with 2 plates and additional bone graft as well as tantalum markers inserted around the symphysis and the SIJ in 2009 (Figure 1). The symphysis was also surgically fixated with a plate, but patient 1 had the plate removed in 2012 (Figure 1). At the time of the present examination patient 1 (age 45) had visible implant failure at the left SIJ. Patient 2 (age 60) continued to experience pain in the operated right SIJ. Both patients showed no certain signs of fusion on radiographs. One of the authors (SMR) did RSA analyses, and CT-RSA analyses were done by a collaborator from Sectra AG (Sweden).

The manuscript complies with the CARE checklist for case reports.

### Patient positioning and provocation

**Figure 2:**
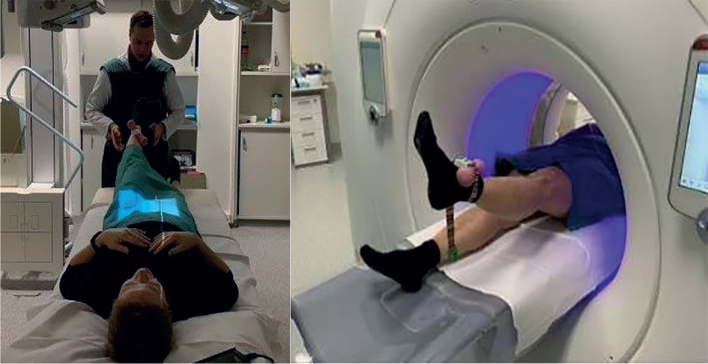
Setup with active straight leg raise during radiostereometric analysis (RSA) and CT-RSA.

The first image of each modality was taken with the patients in the supine position, representing the reference examination. The second image was taken with the leg elevated around 30°. The elevated leg was loaded with 1 kg to induce additional stress and movement in the SIJ (provocation). One examiner supported the leg in the early phase of the elevation maneuver and then let the patient stabilize the leg in this ASLR position. The patient was holding the leg on her own during image acquisition. The ASLR was performed on both sides, and as the leg was loaded with 1 kg, we refer to it as modified ASLR (Figure 2).

### Markings, coordinate systems, and motion analysis

The patients had 5–9 RSA markers (1 mm) present in each segment. The sacrum was regarded as the reference segment. The moving segment was defined by the markers in the posterior ilium and the symphysis, which we named the iliopubis segment. We used RSA software to calculate motion for the center of the iliopubis segment along the coordinate system [4]. Motion was calculated between the sacrum (reference segment) and the left and right iliopubis (moving segment).

The limit for the acceptable condition number was 150 and the mean error was 0.35 mm for the RSA analysis.

### Imaging setup

For conventional marker-based RSA, we used the standard set up of 2 side-by-side X-ray tubes with the X-ray beams angled 40°. The calibration cage 43 (UmRSA Biomedical, Umeå, Sweden) was placed underneath the table. Markers were identified with RSA software from UmRSA Biomedical (Version 6.0) [5].

The imaging parameters for the CT examinations were 150 Sn kVp, effective dose was 213 mAs, rotation time 1.0 s, pitch 0.8, collimation of 192 x 0.6, filter/kernel Br59, slice thickness 0.625 mm, ADMIRE 2 (advanced modeled iterative reconstruction 2) applied (Siemens Force; Siemens Healthineers, Forchheim, Germany).

### CT-RSA and visualized motion

We segmented the surface of the sacrum and the bony structures of the iliopubis with CT-RSA software (CT-IMA, Sectra, Linköping, Sweden). Motion of the segmented iliopubis bone for each side was analyzed against the sacrum between the first and second CT image [4].

The center of rotation was manually moved to the anticipated center of rotation of conventional RSA. Both CT-RSA and RSA are based on a 3-dimensional coordinate system along 3 axes, X, Y, Z, and reported as translation in millimeters and rotation in degrees. The coordinate systems—for RSA is defined by the calibration cage, for CT-RSA as DICOM—were aligned as follows to enable comparison of motion: transverse axis: CT-RSA x = RSA x, sagittal axis CT-RSA y = –RSA z,, and longitudinal axis CT-RSA z = RSA y [4].

### Protocols for calculating precision

We performed a double examination in each patient in the supine position with conventional RSA to calculated precision resulting in 2 double examinations [6].

For CT-RSA, we regarded the sacrum as a stiff non-elastic bone, which allowed us to calculate the precision by comparing motion of the left and right sides of the sacral bone. This movement was calculated between each provocation and compared with the neutral position in the patient, respectively, resulting in 4 double examinations in these 2 patients [**7**]. We refer to this measurement as “intrasegmental precision.” Precision was expressed as mean and SD for RSA and CT-RSA.

### Ethics, registration, funding, and disclosures

The patients signed an informed consent form and agreed to the study and publication of the article. The study was approved by the Regional Committee for Medical Research Ethics (REK Nord 2017/1380) and performed in accordance with the ICMJE Recommendations for the Protection of Research Participants and the Helsinki Declaration, as revised in 2013.

Clinical trials; Movement of the Sacroiliac (SI) Joint, Comparing Conventional RSA with the Sectra Implant Movement Analysis (IMSRA), NCT04752007. ClinicalTrials.gov PRS: Record Summary NCT04752007.

The authors declare that they have no competing interests. The authors received financial support from Helse Vest Norway. Complete disclosure of interest forms according to ICMJE are available on the article page, doi: 10.2340/17453674.2026.45479

## Results

The measured movement is above the precision limits of both methods. CT-RSA and RSA showed comparable precision, ranging from 0.003 to 0.062 mm and –0.05 to 0.02 mm for translation, and 0.01° to 0.03° and –0.10° to 0.04° for rotation, respectively (Table 1). We detected translations in the range of –0.75 to 0.46 mm and rotations between –0.65 and 0.59° using CT-RSA, and with RSA translations in the range of –0.41 to 0.39 and rotations between –0.55 and 2.22 (Table 2). The detected CT-RSA motion is comparable to that of RSA (Table 2). The precision of CT-RSA is slightly better than of RSA (Table 1). In both patients, movement could be measured in the possibly non-fused SIJs. The non-fixated SIJ showed larger movement than the fixated during the modified ASLR. The modified ASLR provocation was successfully performed during CT examinations.

**Table 1:** Precision for radiostereometric analysis (RSA), analyzed between sacrum and iliopubis in neutral double examinations and for CT-RSA left vs right sacrum in neutral and by provocation. The CT-RSA analysis coordinate system has been adjusted to that of RSA for the movement and before the precision calculation

Precision				
Axis	Translation (mm), mean (SD) CT-RSA n = 4	Rotation (°), mean (SD) RSA n = 2	CT-RSA n = 4	RSA n = 2
x	0.00 (0.01)	–0.05 (0.08)	0.03 (0.08)	0.04 (0.18)
y	0.00 (0.06)	0.04 (0.08)	0.01 (0.02)	–0.04 (0.12)
z	0.04 (0.02)	0.02 (0.08)	0.02 (0.07)	–0.10 (0.06)

**Table 2:** Sacroiliac joint (SIJ) movement during active straight leg raise (ASLR) measured using radiostereometric analyses (RSA) and CT-RSA. There is a tendency towards a higher induced movement in the contralateral sacroiliac joint. Patient 1 had the left SIJ, and patient 2 had the right SIJ plate fixated in 2009. The measurable movement of both joints indicated possible pseudarthrosis of the SIJs

Patient		Induced displacement				
Axis		Translation (mm)		Rotation (°)		
Provocation		SIJ side	CT-RSA	RSA	CT-RSA	RSA
Patient 1						
x	ASLR right	R	0.09	0.05	0.47	0.14
		L	0.46	0.39	–0.16	–0.38
	ASLR left	R	–0.30	–0.18	–0.56	–0.39
		L	–0.25	–0.33	0.12	0.26
y	ASLR right	R	–0.41	–0.01	0.10	0.31
		L	–0.01	0.12	0.50	–0.55
	ASLR left	R	0.21	0.05	–0.36	–0.06
		L	–0.23	–0.09	–0.13	0.29
z	ASLR right	R	0.07	–0.06	–0.02	0.03
		L	0.11	0.24	–0.65	0.53
	ASLR left	R	0.09	0.21	0.26	–0.41
Patient 2						
x	ASLR right	R	0.33	0.14	0.36	0.12
		L	0.32	0.38	–0.39	0.10
	ASLR left	R	–0.06	–0.34	–0.02	0.37
		L	–0.03	–0.18	0.59	2.22
y	ASLR right	R	–0.47	0.07	0.24	–0.02
		L	0.30	0.26	0.43	–0.92
	ASLR left	R	–0.05	–0.06	–0.11	–0.01
		L	–0.75	0.19	–0.08	0.42
z	ASLR right	R	–0.07	–0.14	–0.15	0.31
		L	0.30	0.33	–0.37	–0.87
	ASLR left	R	–0.11	–0.34	0.24	–0.43
		L	0.09	–0.41	0.24	0.45

**Figure 3:**
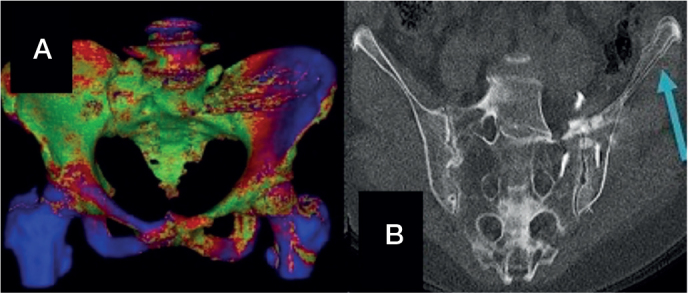
(A) CT-radiostereometric analysis (CT-RSA) motion map by type and side of provocation in patient 1. Red—areas of expected motion, blue—areas with most motion, green—with little or no motion. In the present image, an active straight leg raise (ASLR) (right) leads to more motion in the contralateral iliopubis. (B) Flipbook animation in patient 1 with pseudarthrosis in the left sacroiliac joint. An overlap of the neutral and ASLR CT-RSA images showing the motion of the left ileum (arrow).

The induced motion is reported numerically (Table 2) and visualized as a motion heat map or optionally also as a flipbook animation (Figure 3).

The mean effective dose (ED) for each examination/total ED for each patient during RSA was 0.8/2.4 mSv (4 examinations/patient) and for CT-RSA 6.2/18.6 mSv (3 examinations/patient). ED was 7.8 times higher for CT-RSA than for RSA.

## Discussion

We aimed to clarify whether CT-RSA can measure in vivo movement in the SIJ during a modified ASLR. We found movement in the SIJs bilaterally measured with both CT-RSA and RSA. This feasibility study demonstrates the possibility to detect motion and failed fusion of the SIJ with CT-RSA during a modified ASLR test to induce a displacement. This has not been described in the literature before. Olivecrona et al. [8] have shown movement of the SIJ in a fig-4-position/provocation with CT-RSA in 12 patients. In that study a translation (mm) of 0.18 (SD 0.14) (95% confidence interval [CI] 0.08–0.28) and rotation 0.20° (SD 0.12) (CI 0.11–0.28) within the sacrum was detected. This was described as viscoelastic properties of bone or error in measurement. They also described an SIJ translation of 0.57 mm (SD 0.25) (CI 0.39–0.75), and a rotation of 0.57° (0.20) (CI 0.43–0.71) in non-fused joints. We assume zero motion in a fused joint. In a pseudoarthrosis joint we expect motion, and the measured motion in our study shows similar movement in the pseudarthrosis joints and native joints. Some movement might be explained by the elasticity in the bony structures. In the visual interpretation the movement of the pseudarthrosis is clearly visible.

A challenge is to induce movement in the SIJ because of its ligamentous and bony anatomy. Modified ASLR has the potential to do so [9], but the degree of dislocation is low [5]. In an RSA study, Kibsgård et al. found a backward rotation of only 0.8° and an inward tilt of 0.3° during the ASLR [5]. This motion is comparable to our findings for CT-RSA and RSA.

Precision is the degree to which a test will repeat the same value and, therefore, is of interest when introducing a new method. Due to the radiation exposure, we did not perform any CT double examinations but calculated precision intersegmentally, as described earlier by Poulsen et al. [7]. We compared precision of zero motion of 2 rigid bodies within a segment. This allows us to reduce the total irradiation dose as provocation examinations always include 2 images anyway and therefore can be used to calculate precision.

Precision for CT-RSA with bone models was comparable to or slightly better than that for marker-based RSA (see Table 1). This is in accordance with earlier studies comparing the precision of RSA and CT-RSA in other joints [2,3,10-12]. Recently, Angelomenos et al. [13] compared the precision of low-dose CT-RSA vs marker-based RSA for early acetabular cup migrations and found a similar precision in the range of 0.06–0.13 mm (CT-RSA) and 0.06–0.15 mm for translation, and 0.23–0.35° (CT-RSA) and 0.21–0.63° (RSA) for rotation. Nevertheless, our precision data should be interpreted with caution because of the low number of cases.

We detected a translation in the range of –0.75 to 0.46 mm and –0.65 to 0.59° for rotation of sacrum vs the iliopubis using the CT-RSA. The movement detected was above the precision limits for CT-RSA. The translation and rotation in the SIJ were comparable to earlier RSA studies [5,14]. The detected rotation for RSA has 1 rotation that is quite high: 2.22°. This might be caused by the out-of-plane rotation around the x-axes or by inferior marker spreading caused by the small area of the SI joint. Provocation in joints is difficult to standardize in different clinical settings. Although the provocation was performed in the same way in the CT-RSA and RSA settings, the consecutive provocations never cause exactly the same joint movements through the muscle forces.

A new modality of CT-RSA compared with conventional marker-based RSA is that the software allows direct visualization of the movement. During the workflow of CT-RSA by Sectra, a heatmap shows the different degrees of motion (see Figure 3A) illustrated by different coloring. A flipbook animation of the CT images can also be created by switching from neutral to the provocation position (see Figure 3B) of the bone.

We followed the CT-RSA developer’s (Sectra AG) recommendation when performing the CT examinations, which at that time did provide low-dose protocols. The effective dose for CT-RSA was 7.8 times higher than that of RSA (mean 6.2/0.8 mSv) in our 2 patients. The irradiation dose must be reduced for further clinical use [4]. Several studies in other joints show that this is possible without losing precision [8,11,15]. In a clinical setting a single provocation of the painful side will reduce the radiation dose by one-third compared with our bilateral provocation. A clear advantage of CT-RSA is that it can be performed without the use of bony markers and therefore might be useful in clinical practice.

### Limitations

Possible inaccuracies of the center of motion calculated by the software for RSA and CT-RSA might be responsible for some numeric differences. We present only 2 patients in this feasibility study but Reiser et al. [16] also only relies on 3 patients to present a provocation of the wrist and the same CT analysis method. Further, the precision measurements were performed differently. Nevertheless our range of precision concurs with findings in the literature and the intrasegmental precision method has not previously been applied to the sacrum. Our 2 patients with sacroiliac pain may have exhibited variable responses during provocation tests. No other patient-related limitations were noted.

### Conclusion

CT-RSA can be used to visualize and quantify movement of the SIJ during a modified ASLR and has possibly comparable precision to that of conventional (marker-based) RSA. The irradiation dose must be reduced for further clinical use. CT-RSA offers the benefit of visualization of movement, allows intrasegmental precision measurements within 1 bone, and can be performed without the use of bony markers. This feasibility study adds knowledge concerning CT-RSA as a new diagnostic tool for further research on SIJ pathology.

## Notes

VBH contributed to the examinations, interpreted the patient data, provided analysis, and was a significant contributor to the manuscript. AS contributed to the examinations and interpretation of the images performed and contributed to the manuscript. CH contributed to the manuscript. SMR contributed to the examinations, interpreted the patient data, provided analysis, and was a significant contributor to the manuscript.

The authors would like to express their gratitude to Mona Risdal, radiographer at Oslo University Hospital, for performing the RSA and CT-RSA images.

Handling co-editor: Ivan Hvid

*Acta* thanks Bengt Mjöberg and Marc J Nieuwenhuijse for help with peer review of this manuscript.
